# State- and County-Level Social Determinants of Health-Adjusted Life Expectancy

**DOI:** 10.1093/geroni/igaf122.4405

**Published:** 2025-12-31

**Authors:** Elizabeth Davis, Nasim Ferdows, Daniel Kim

**Affiliations:** Northeastern University, Boston, Massachusetts, United States; Northeastern University, Boston, Massachusetts, United States; School of Community Health and Behavioral Sciences, Bouvé College of Health Sciences, Northeastern University, Boston, Massachusetts, United States

## Abstract

In the United States, health-adjusted life expectancy (HALE) declined by 0.3 years between 2009 and 2019, which may reflect increased deaths related to drug overdose, suicide, and alcohol. To combat the rise in such “deaths of despair,” it is critical to identify their root causes including social and economic determinants and address them through corresponding policies. We obtained county-level HALE from 2009 and 2019 through the Global Burden of Diseases, Injuries, and Risk Factors Study. Using state fixed effects models and these panel data, we explored multiple social and economic determinants of HALE simultaneously including the state-level percentage in poverty, gender pay gap ratio, violent crime rate, tax burden, earned income tax credit rate, income inequality, welfare spending, and education spending; and county-level social capital, debt-to-income ratio, and racial segregation. We found several of these factors to be positively associated with HALE including a higher state tax burden and earned income tax credit rate as well as county-level median household income, social capital, and debt-to-income ratio. Moreover, we determined other factors to be inversely linked to HALE including state-level median income, the percentage in poverty, percentage unemployed, and income inequality level; as well as the county-level percentage unemployed. Overall, our findings suggest a variety of specific social and economic determinants of HALE that may help to inform future interventions and policies in order to promote healthy aging and advance healthy longevity among Americans.

